# Theoretical and Numerical Analysis of Impact Forces on Blocking Piles Within Embankment Breaches Using Flow Velocity Signals

**DOI:** 10.3390/s25113333

**Published:** 2025-05-26

**Authors:** Xing-Huai Huang, Yu Fang, Sheng-Yu Chang, Ying-Qing Guo

**Affiliations:** 1 China-Pakistan Belt and Road Joint Laboratory on Smart Disaster Prevention of Major Infrastructures, Southeast University, Nanjing 211189, China; huangxh@seu.edu.cn (X.-H.H.); 220211175@seu.edu.cn (Y.F.); 220241525@seu.edu.cn (S.-Y.C.); 2 Mechanical and Electronic Engineering College, Nanjing Forestry University, Nanjing 210037, China

**Keywords:** embankment pile plugging, water flow impact force, numerical simulation, Morison equation method, flow velocity signals

## Abstract

In the realm of structural health monitoring (SHM) and smart disaster prevention, accurately assessing the impact forces on emergency structures during natural disasters is crucial for a timely and effective response. Therefore, a theoretical method for the water flow impact force on embankment breach piles was established by combining the numerical model of breach hydraulics with the Morison equation. To assess the accuracy and validity of the proposed theoretical calculation method, a 3D finite element model considering the coupling effect of water flow and pile arrangement was established, and the effects of flow velocity, water depth, and other factors on the force of the plugging structure were studied. A comparative analysis was conducted and indicated that the Morison equation method based on the flow velocity signals can calculate the impact force of the structure within a certain error range when the value of drag force coefficient $C_{D}$ is set to 1.0 and the value of inertia force coefficient $C_{M}$ is set to 2.0, providing a reference for emergency plugging decisions for embankment breaches. The findings provide essential theoretical references for data-driven emergency plugging decisions, thereby enhancing the effectiveness of smart disaster prevention strategies for embankment breaches.

## 1. Introduction

Embankment engineering plays an important role in irrigation, power generation, navigation, and other fields. However, the breach of embankments caused by floods during the flood season seriously threatens people’s lives and property safety. Emergency plugging by pile placement at the breach can protect downstream land and houses from continuous flood damage and is a committed strategy to mitigate disaster-related losses. However, during the blocking of breaches, the impact forces generated by high-velocity flow can damage the blocking pile structure, leading to safety hazards during construction and affecting the efficiency of the blockage. If the water flow impact forces can be quickly estimated based on the site flow velocity and the form of the pile structure, a rapid safety assessment of the piling construction plan can be made, thereby guiding the blockage construction process to be safer and more efficient.

The current research on the flow force of dam breaches mainly focuses on the impact of dam breach flow on downstream structures and is mainly conducted through experiments and numerical simulations [1,2,3,4,5,6,7,8,9,10]. Lobovsky [11] et al. conducted experimental research on the breach flow on a horizontal dry bed, calculating the pressure load and dynamic impact of the breach wave on the downstream vertical wall; Kocaman [12] studied the effect of breach shock waves on downstream vertical walls through experimental testing and CFD simulation and compared and analyzed the simulation results of Reynolds averaged N-S equation and shallow water equation; Kuswandi [13] studied the impact force of dam break waves on vertical cylinders and investigated the effects of reservoir length, depth, and downstream water depth on the impact force; Dumergue [14] conducted extensive numerical simulations to investigate the effects of downstream depth and obstacle position on the impact force of dam break waves on downstream obstacles. Therefore, there is currently a lack of research on the force of the plugging structure for embankment breaches.

With the development of computer technology, three-dimensional finite element simulation can more accurately obtain the characteristics of breach water flow and the impact force of pile flow. Hien [15] simulated the impact of breach waves on isolated buildings or building clusters using two- and three-dimensional numerical models and studied the effects of initial water level and breach width on the peak impact force. However, when making emergency plugging decisions for embankment breaches, it is obviously unrealistic to use finite element simulation. Therefore, there is an urgent need to study the numerical calculation method of the water flow impact force of the embankment breach plugging structure. Some scholars calculate the water flow impact load through the Morison equation [16,17,18,19,20,21,22,23,24,25,26,27,28]. Vengatsan [29] studied the effect of wave forces on rectangular cylinders through experiments and calculated the hydrodynamic coefficients. In 2006, Chen [30] et al. measured the impact pressure of the Qiantang River surge and proposed a semi empirical and semi theoretical method to calculate the water flow impact force and proposed a formula for the water flow impact coefficient; He [31] studied the hydrodynamic and total wave loads of piles under different layout schemes through numerical simulation, which is consistent with the results of Morison’s equation. Saincher [32] studied the combined hydrodynamic loads of nonlinear waves and uniform flow on a monopile cylindrical marine structure. Using the Morison theory and fully nonlinear potential theory, the interaction between unbroken and broken waves with the structure was analyzed. The experimental results indicate that regardless of the wave properties and drag velocity, the combined effect of wave force and uniform flow is linear when wave current interaction is excluded. Xin [33] studied the coupling effect of irregular waves and water flow on the hydrodynamic loads of slender structures and the influence of structural impact loads. However, the calculation of the Morison equation requires determining appropriate drag and inertia force coefficients, as well as obtaining the velocity, acceleration, and surface characteristics of the water flow. However, much of the research focuses on CFD, FEM, or other numerical methods, which, while capable of providing high-accuracy solutions, require significant computational time. This makes them unsuitable for the emergency plugging of breaches. Therefore, it is essential to quickly calculate the water flow impact force using theoretical methods, which can help guide the blockage construction process to be safer and more efficient.

This paper mainly focuses on the research of the force on the plugging structure of embankment breach, aiming to construct a numerical calculation method for the water flow impact force of the plugging structure of embankment breach, and provide reference for emergency plugging decision-making. To achieve this, a two-dimensional shallow water equation was established on a structured grid, and a Godunov scheme hydraulic model was constructed. At the same time, a three-dimensional finite element model was used to analyze the influence of pile arrangement on the water flow development of the embankment breach. The influence of river flow velocity, water depth, pile arrangement method, inclination, etc. on the water flow impact force of the pile arrangement was studied, and a more detailed study was conducted on the force of the plugging structure.

## 2. Numerical Calculation Method for the Impact Force of Embankment Breach Flow on Plugging Structures

### 2.1. Two-Dimensional Shallow Water Equation

The Navier Stokes equation in three-dimensional fluid dynamics describes the motion and changes of fluids in time and three-dimensional space, ignoring the viscosity and heat conduction in the N-S equation. The mass conservation and energy conservation equations are combined to obtain the following Euler equation in Cartesian coordinates:

(1) $$\left\{\begin{array}{c} \tau_{x}=\lambda \left(\frac{\partial u}{\partial x}+\frac{\partial v}{\partial y}+\frac{\partial w}{\partial z}\right)+2\mu \frac{\partial u}{\partial x}\\ \tau_{y}=\lambda \left(\frac{\partial u}{\partial x}+\frac{\partial v}{\partial y}+\frac{\partial w}{\partial z}\right)+2\mu \frac{\partial v}{\partial y}\\ \tau_{z}=\lambda \left(\frac{\partial u}{\partial x}+\frac{\partial v}{\partial y}+\frac{\partial w}{\partial z}\right)+2\mu \frac{\partial w}{\partial z} \end{array}\right.,\left\{\begin{array}{c} \tau_{xy}=\mu \left(\frac{\partial u}{\partial y}+\frac{\partial v}{\partial x}\right)\\ \tau_{xz}=\mu \left(\frac{\partial u}{\partial z}+\frac{\partial w}{\partial x}\right)\\ \tau_{yz}=\mu \left(\frac{\partial v}{\partial z}+\frac{\partial w}{\partial y}\right) \end{array}\right.,\lambda =-\frac{2}{3}\mu$$

in which ρ is the density of the fluid, *t* is time, *p* is pressure, *τ* is a force tensor, and *f* is a volumetric force equal to gravity. *U* is the solution vector of the equation, *F*, *G*, and *H* are fluxes, *u*, *v*, and *w* are velocities in three directions, *μ* is the molecular viscosity coefficient, and λ is the second viscosity coefficient.

Simplifying the three-dimensional N-S equation into a two-dimensional shallow water equation is a common approximation method, especially suitable for describing fluid motion with a horizontal scale far greater than the vertical scale. The width of a dike breach is generally much greater than the depth of a river, and the water flow can generally be considered incompressible. The density remains constant in space and time. The evolution of the water flow at a dike breach mainly occurs in the plane, and the velocity changes slowly in the vertical direction. The two-dimensional shallow water equation is obtained by integrating and simplifying along the water depth direction:

(2) $$\left\{\begin{array}{c} S_{ox}=-\frac{\partial Z_{b}}{\partial x}\\ S_{oy}=-\frac{\partial Z_{b}}{\partial y} \end{array}\right.$$

(3) $$\left\{\begin{array}{c} S_{fx}=n^{2}u\sqrt{u^{2}+v^{2}}h^{-\frac{4}{3}}\\ S_{fy}=n^{2}v\sqrt{u^{2}+v^{2}}h^{-\frac{4}{3}} \end{array}\right.$$

where *S_ox_* and *S_oy_* denote the bottom slope terms in the *x* and *y* directions, respectively. *Z_b_* represents the bottom elevation, while *S_fx_* and *S_fy_* represent the frictional slope in the *x* and *y* directions, respectively. *n* represents the Manning roughness coefficient, and *h* represents the height of the water surface. 

The hydrodynamic force per unit length calculated according to the Morison equation is:

(4) $$f=f_{D}+f_{I}=\frac{1}{2}C_{D}\rho Au_{x}\left|u_{x}\right|+C_{M}\rho V_{0}\frac{du_{x}}{dt}$$

Various countries have also specified the values of $C_{D}$ and $C_{M}$ in their regulations, as listed in Table 1. After multiple simulation studies, it has been found that under the condition of embankment breach, a $C_{D}$ value of 1.0 and a $C_{M}$ value of 2.0 can better predict the water flow impact force at the breach.

In the two-dimensional model, in order to accurately simulate the interaction between fluid and structure, it is necessary to integrate the relevant input parameters with the Morison equation. The input parameters of the two-dimensional model usually include the velocity, density, and viscosity coefficient of the fluid, as well as the geometric parameters of the structure, such as shape, size, and position.

In the integration process, according to the flow field information provided by the two-dimensional model, the fluid velocity distribution acting on the surface of the structure is determined. Then the inertia force and drag force acting on the structure are calculated according to the Morison equation. The inertial force is determined by considering the volume of the structure and the fluid acceleration. The drag force is calculated according to the shape coefficient of the structure, the square of the fluid velocity, and the relevant drag coefficient.

These forces calculated by the Morison equation are introduced into the motion equation or force balance equation of the two-dimensional model as external force terms. In this way, the two-dimensional model can comprehensively consider the dynamic characteristics of the fluid and the interaction between the structure and the fluid so as to more accurately simulate and analyze the response of the structure in the fluid environment.

### 2.2. Solution of the 2D Shallow Water Equations Based on the Godunov Scheme

#### 2.2.1. Grid Division and Boundary Conditions

**Figure 1:**
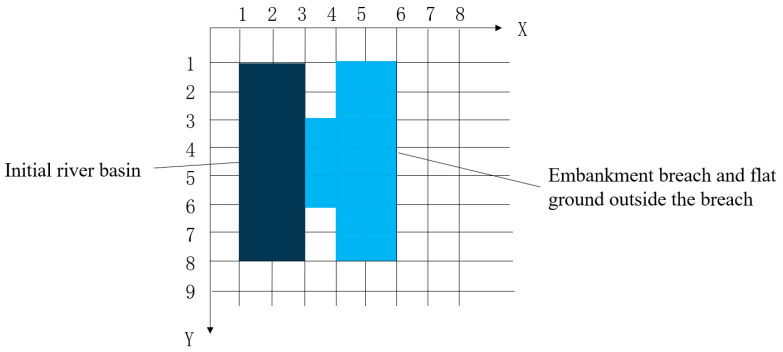
Schematic diagram of two-dimensional computational grid division.

The finite volume method is adept at handling non-uniform and unstructured grids, ensuring the local quantities within a single volume are locally conserved, and it offers superior quality and energy conservation characteristics compared to the finite difference method. Two-dimensional mathematical models for fluid flow can utilize structured grids. For breaches with a regular shape, employing a rectangular grid facilitates clear identification of the relationships between grid cells, simplifying program design and enhancing computational efficiency. If the breach has an irregular shape, it may be necessary to adopt a partial triangular grid to accommodate complex boundary conditions. The two-dimensional mesh division used in this study is shown in Figure 1.

During the computational process, the coordinates, time, and flux values in both directions for each control volume are represented as function values by using a multidimensional array. The fluxes at all boundaries also need to be recorded and stored, which can be efficiently managed using a categorized approach. The grid is progressively refined from the river region towards the breach and the adjacent plane areas. 

The finite volume method employs average integration within each element to maintain the physical quantities constant within each element. The set of control elements within the solution domain is assembled into a sequence of piecewise functions. At the interface between elements, the physical quantities being computed may pose a local Riemann problem due to fracture formation, necessitating the solution of the Riemann problem.

The grid generation strategy in this paper uses fluent meshing to generate the grid, which is completed by four steps: geometric simplification, region division, grid type selection, and grid density control.

**Figure 2:**
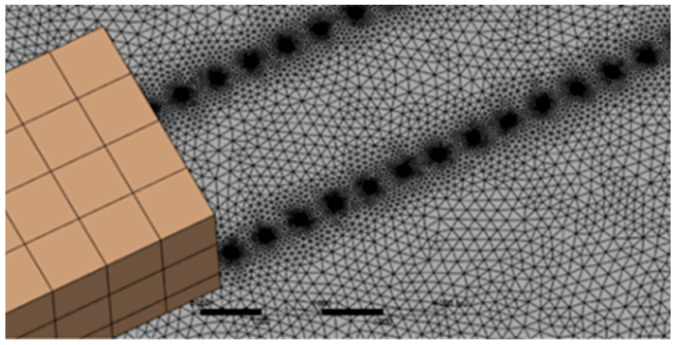
Model grid generation diagram.

When meshing, the grids with different sizes were selected in turn, and the results were verified. The calculated flow impact force was within the error range, so the grid size of 0.5 m was finally selected for calculation. Grid division is shown in Figure 2.

When processing the boundary layer, select the boundary layers tab in the mesh setup, add the wall surface that needs to generate the boundary layer, and then set the relevant parameters. The K-ε model is selected and the wall parameters are set for numerical simulation analysis.

#### 2.2.2. Solution of Riemann’s Problem

The solution to Riemann’s problem can be divided into two methods: exact solution and approximate solution. Exact solution refers to completely solving the Riemann problem and obtaining accurate flux values for each point on the interface. The approximate solution is to use some numerical methods, such as Godunov scheme or other methods based on numerical flux calculation, to approximate the Riemann problem and obtain approximate flux values, which has higher computational efficiency.

The commonly used Riemann approximate solutions include the Roe scheme and the HLL scheme. The basic idea of the Roe scheme is to approximate nonlinear problems through linearization, thereby simplifying the solving process by linearizing nonlinear problems into a series of local Riemann problems. In the Roe scheme, feature variables and feature velocities are introduced, and the numerical flux expression is:

(5) $$F_{j+\frac{1}{2}}=\frac{1}{2}{(F}_{j}+F_{j+1})-\frac{1}{2}\sum_{i=1}^{3}\tilde{d_{i}}\left|\tilde{\lambda_{i}}\right|\tilde{R_{i}}$$

where $\tilde{\lambda_{i}}$ is the three eigenvalues. The specific expression form is as follows:

(6) $$\tilde{u}=\frac{\sqrt{h_{L}}u_{L}+\sqrt{h_{R}}u_{R}}{\sqrt{h_{L}}+\sqrt{h_{R}}},\tilde{v}=\frac{\sqrt{h_{L}}v_{L}+\sqrt{h_{R}}u_{R}}{\sqrt{h_{L}}+\sqrt{h_{R}}},\tilde{c}=\sqrt{\frac{{g(h}_{L}+h_{R})}{2}}$$

$where\;\tilde{u}$, $\tilde{v}$ is the average value of Roe, and $\tilde{c}$ is the average wave velocity. If one side of the unit interface is a dry unit, then:

(7) $$\tilde{u}=\frac{u_{R}+u_{L}}{2},\tilde{v}=\frac{u_{R}+v_{L}}{2}$$

Perform feature decomposition on the average Jacobian matrix of Roe:

(8) $$\left[\begin{array}{c} \tilde{d_{1}}\\ \tilde{d_{2}}\\ \tilde{d_{3}} \end{array}\right]=\left[\begin{array}{c} \tilde{L_{1}}\\ \tilde{L_{2}}\\ \tilde{L_{3}} \end{array}\right]∆U=\left[\begin{array}{c} \tilde{L_{1}}\\ \tilde{L_{2}}\\ \tilde{L_{3}} \end{array}\right]\left(U_{R}-U_{L}\right)=\left[\begin{array}{c} \frac{1}{2}(∆h-\frac{\sqrt{h_{L}h_{R}}}{\tilde{c}}∆u)\\ \sqrt{h_{L}h_{R}}∆v\\ \frac{1}{2}(∆h+\frac{\sqrt{h_{L}h_{R}}}{\tilde{c}}∆u) \end{array}\right]$$

where $U_{L}$ and $U_{R}$ are invariant physical quantities on the left and right sides of the unit interface, respectively.

The fundamental concept behind the HLL scheme is to disassemble the set of conservation equations into two Riemann problems, focusing on the flux exchange between adjacent control volumes on either side. These two Riemann problems mirror the flow patterns on either side, and by addressing them, the respective left and right fluxes are derived. These fluxes are then fused together using linear interpolation, yielding the overall interface flux. The numerical flux in the HLL scheme is expressed as:

(9) $$h_{*}=\frac{1}{g}{\left[\frac{1}{4}(u_{L}-u_{R}+2\sqrt{gh_{L}}+2\sqrt{gh_{R}})\right]}^{2}$$

(10) $$u_{*}=\frac{1}{2}\left(u_{L}+u_{R}\right)+\sqrt{gh_{L}}-\sqrt{gh_{R}}$$

(11) $$S_{L}=max(u_{L}-\sqrt{gh_{L}},u_{*}-\sqrt{gh_{*}})$$

(12) $$S_{R}=max(u_{R}-\sqrt{gh_{R}},u_{*}+\sqrt{gh_{*}})$$

where $F_{L}$ and $F_{R}$ represent the conserved flux of the left and right control bodies, respectively, $U_{L}$ and $U_{R}$ represent the conserved flux of the left and right control bodies, respectively, and $S_{L}$ and $S_{R}$ represent the maximum eigenvalues of the left and right control bodies, respectively.

However, the HLL format also has some limitations and drawbacks, such as insufficient accuracy in handling strong shock waves and high-speed flows, which require improvement and optimization in combination with other numerical methods. Therefore, this article calculates based on the Roe method.

#### 2.2.3. Second-Order Correction of the Godunov Scheme

**Figure 3:**
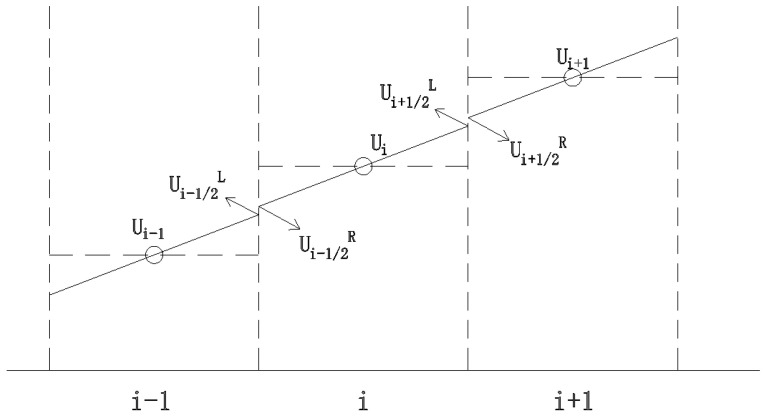
MUSCL method for reconstructing the left and right interfaces of grids. In the figure, x and y axes represent the grid numbers, $U_{i}$ represent means water flow velocity at point *i*.

**Figure 4:**
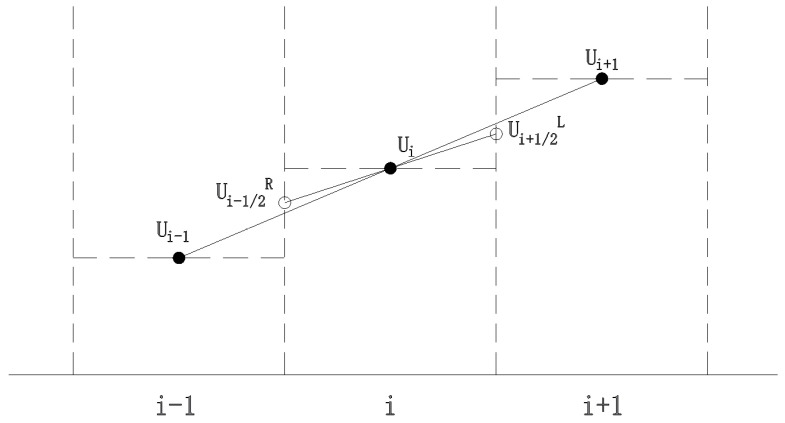
Schematic diagram of TVD restriction function. In the figure, x and y axes represent the grid numbers, $U_{i}$ represent means water flow velocity at point *i*.

When the physical quantity within the control unit remains constant, the computational precision is only of first order. To accurately reflect local variations, a second-order correction is necessary to enhance the accuracy of the numerical solutions. This study reconstructs the conserved variables on both sides of the unit using the MUSCL method, as depicted in Figure 3. By substituting the reconstructed values for $U_{i}$ and $U_{i+1}$ on either side, we can elevate the precision to second order. However, regions with significant water surface gradients may result in spurious numerical fluctuations. To mitigate such fluctuations, the TVD method, as illustrated in Figure 4, is introduced to maintain the total variation during numerical calculations, thereby preventing artificial oscillations in the numerical solutions, which is particularly apt for the breach discontinuity scenario addressed in this paper.

The TVD scheme with the introduction of restriction functions is formulated as follows:

(13) $$U_{i+1/2}^{L}=U_{i}+\frac{1}{2}\varphi (U_{i+1}-U_{i},U_{i}-U_{i-1})$$

where φ for the limiter function, Roe’s minmod limiter is used in this article, which can effectively handle severe changes such as shock waves and discontinuities and improve the stability and accuracy of numerical calculations. The minmod expression is as follows:

(14) $$\varphi \left(a,b\right)=minmod\left(a,b\right)=\left\{\begin{array}{c} 0\;\;\;\;\;\;\;\;\;\;\;\;\;\;\;\;\;\;\;\;\;\;if\;a<0\;\mathrm{or}\;b<0\\ \min\left(a,b\right)\;\;\;\;\;\;if\;a>0\;\mathrm{and}\;b>0 \end{array}\right.$$

#### 2.2.4. MATLAB Calculation Method for Hydraulic Characteristics of Embankment Breach

The calculation program for the development of water flow in embankment breach is written using MATLAB 2019R. Under the given initial conditions of the river, breach, and water flow, it can calculate the water depth and flow velocity at any position of the breach at any time. The writing method of MATLAB is as follows:

1. Establish parameter models for river channels and breaches. Determine the grid size and store the boundary coordinates and center coordinates of each grid to determine the total calculation time *T*.
2. Set the number of CFLs (Courant numbers). The CFL number is used to control the selection of time steps in numerical solutions to ensure the stability and convergence of numerical solutions. The convergence conditions required for the calculation of 2D shallow water equations are [38]:
  (15) $$N_{CFL}=\frac{max(\sqrt{gh}+\sqrt{u^{2}+v^{2}})\Delta t}{\Delta x/2}\leq 1$$
3. where *h* is the initial depth of the river, u and v are the velocities in the *X* and *Y* directions, Δx is the grid width, where the grid sizes in both directions are the same, and Δt is the time step size, where the value of $N_{CFL}$ is 0.8. Determine the time step size Δt based on $N_{CFL}$.
4. Establish a computational domain. Establish control parameters such as *u*, *v*, *h* for each grid, and initialize them. Set initial flow velocity, river depth, flat height outside of the breach, breach width, and other initial parameter conditions for the river and breach.
5. Calculate the flux of the unit X interface, Y interface, and boundary based on Roe format. Then, calculate new *U*, *F*, *G*, *h*, *u*, *v* values, and continuously advance the time step until the total calculation time *T* is reached, and end the operation.
6. During the calculation process, continuously draw the velocity and water depth values of the target cross-section at each moment in the *X* and *Y* coordinates.

## 3. Numerical Simulation for the Impact Force of Embankment Breach Flow on Plugging Structures

The previous chapter numerically solved the 2D shallow water equation based on the Godunov scheme, and based on the numerical model of water flow, derived the theoretical calculation formula of water flow impact force through the Morison equation. However, the two-dimensional shallow water equation has been simplified to a certain extent, and the calculated results are not accurate enough. Unstructured grids need to be introduced for complex terrains, making it difficult to process. When using the Morison equation theory to calculate the impact force of water flow, the values of $C_{D}$ and $C_{M}$ have different errors under different working conditions, and the calculation results will also produce certain errors. Based on this, a more refined three-dimensional breach model is used to study the influence of plugging structures on the characteristics of breach water flow, deduce the development trend of breach water flow, and study the impact force characteristics of breach pile flow under different factors.

### 3.1. Establishment of Finite Element Model for 3D Embankment Breach Water Flow Prediction

This study mainly focuses on the hydraulic characteristics of the water flow at the breach of the embankment after instantaneous breach, using a rectangular channel and ignoring the slope. The model channel is 70 m long and 20 m wide, with a breach width of 15–30 m in the figure. The breach occurs at a distance of 35 m from the upstream entrance, with a dike width of 4 m and a height of 5 m. When analyzing the characteristics of the breach water flow, the initial channel speed is set to 3 m/s, the river depth is 3 m, and the outer side of the breach is flat without any obstruction. 

**Figure 5:**
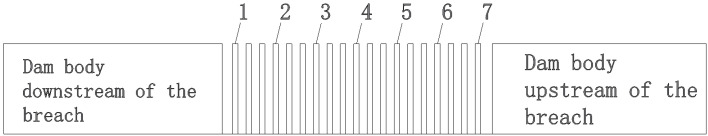
Pile plugging structure and numbering.

The diameter of the row piles is 0.3 m, the wall thickness is 0.01 m, the distance between the pile centers is 0.75 m, the free length above the mud surface is 5 m, the standard model water flow depth is 3 m, the river flow velocity is 3 m/s, the width of the breach is 15 m, and the row piles are driven near the X3 section of the breach. To understand the force situation of steel pipe piles at different positions, seven steel pipe piles were selected at equal intervals along the Y-axis direction for analysis, numbered 1–7, as shown in Figure 5.

When modeling in Fluent, due to the initial water flow distribution in the river, a VOF multiphase flow model needs to be used, where air is the main phase and water flow is the secondary phase. VOF is a pressure-based water flow model that uses the finite volume method to calculate the volume fraction of the target fluid in each grid of the watershed, in order to perform numerical analysis and track the interface of each phase. VOF can also capture free liquid surface flow well in strongly discontinuous irregular water flow, making it suitable for breach models. The RNG model in k−ε is selected, which is more sensitive to the effects of rapid strain and streamline curvature compared to the standard model, and is suitable for analyzing breach water flow.

**Figure 6:**
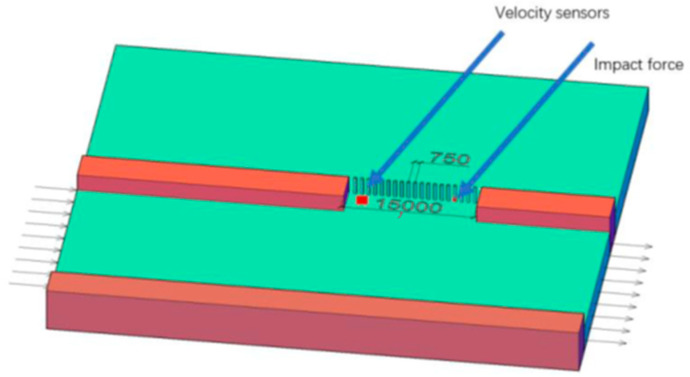
Three-dimensional model and initial flow distribution of rivers in Fluent. The green color means water and the brown color represents embankment.

The model is used for transient analysis to capture the changes in water flow characteristics over time. The velocity inlet is located on the upstream inlet side, and its value is the initial river velocity. The downstream is all under pressure boundary conditions, with a pressure value of 0, indicating free flow. The top of the model is under pressure boundary conditions, with a pressure value of 1 standard atmospheric pressure. Other boundary conditions, such as the dam, are set as walls. After initialization, set the initial water flow distribution in the river channel through the patch, as shown in Figure 6.

### 3.2. Simulation of Water Flow Development During the Process of Water Flow Impact Plugging Structure

**Figure 7:**
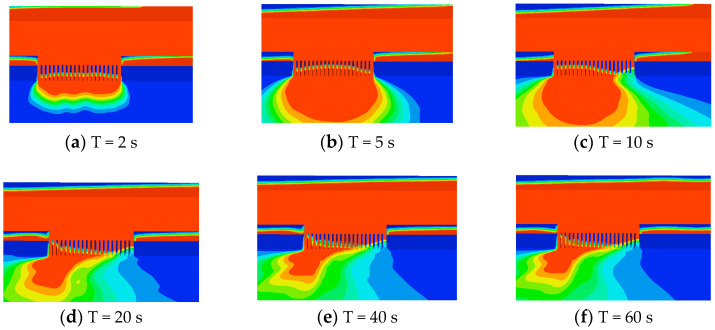
The development process of water flow through plugging structures.

The pile plugging structure has a significant impact on the water flow, and the development of the breach water flow is shown in Figure 7. In the initial stage, the water flow in the original river channel experienced a drop. From the graph, the water flow at the breach flowed through the plugging structure at T = 2 s. Due to structural obstruction, the water flow surged upwards, and the water surface line showed a slight sharp rise. After the water flow passed through the row piles, it sharply decreased and then gradually decreased steadily. At this time, the water depth of the row piles was the same, and there was no significant difference between the steel pipe piles. At T = 5 s, the water level near the original channel breach gradually decreases, and the average depth of water flow inside the breach continuously increases, gradually showing a trend of low water level at both ends and high water level in the middle, as shown in Figure 7b.

**Figure 8:**
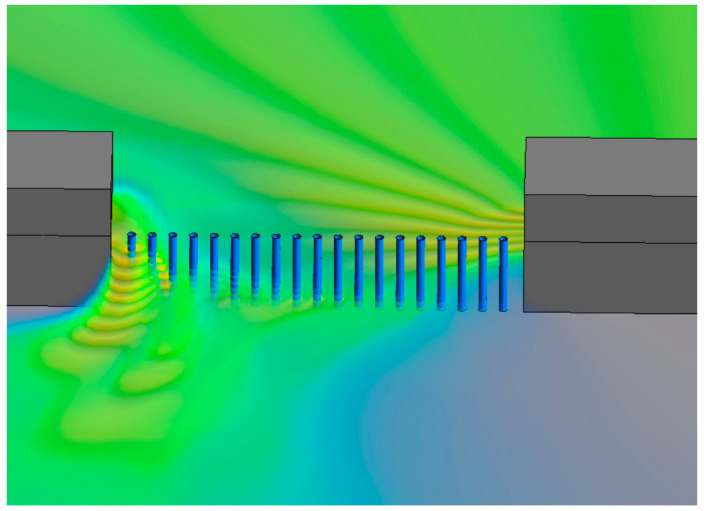
The volume rendering of water flow through plugging structures when T = 60 s.

As time goes on, under the influence of the initial flow velocity of the river, the water flow at the breach began to accumulate downstream. From Figure 7c, it can be seen that the water flow height of the downstream plugging structure is significantly higher than that of the upstream. This trend gradually intensified over time, and the water flow accumulated downstream of the breach. From Figure 7d–f, the water flow maintains this trend and gradually enters a steady state. Figure 8 is the schematic diagram of single-row pile breach water flowing through steel pipe piles when t = 60 s. The degree of water accumulation downstream of the breach depends on the original river flow velocity, and the higher the flow velocity, the higher the degree of accumulation.

### 3.3. Analysis of Water Flow Impact Process on Single-Row Steel Pipe Piles

**Figure 9:**
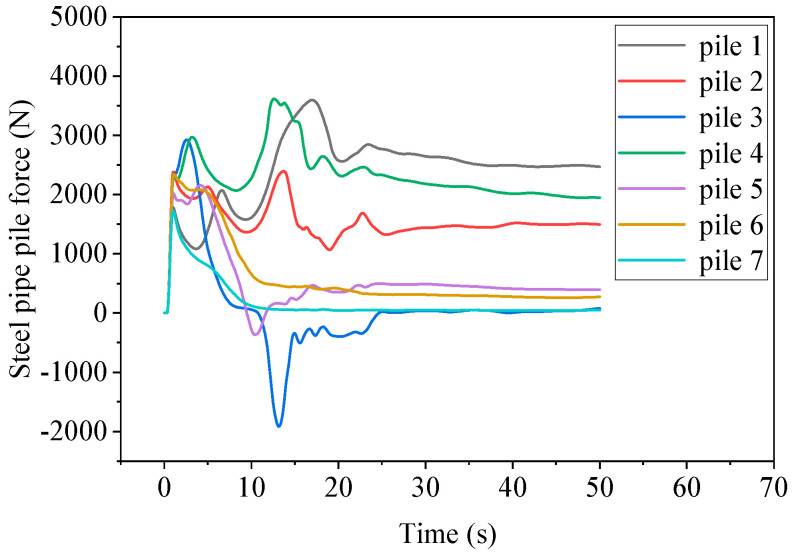
Dynamic time history curve of steel pipe piles 1–7.

From Figure 9, the pile arrangement undergoes complex impact force changes from the initial stage to the steady-state stage. In the initial stage, the water flow at the breach has not yet reached the velocity in the *Y* direction. The water flow at the breach falls along the *X* direction and has contact with various rows of piles simultaneously. All steel pipe piles experience an increase in force, reaching the first peak. As the development trend of the water flow at the breach is high in the middle and low on both sides, the peak values of steel pipe piles 2, 3, 4, 5, and 6 are significantly higher than those of piles 1 and 7. 

After the first wave of breach water flow passes through, the force on row piles gradually decreases, and then the flow velocity of the river gradually increases to 3 m/s. At the same time, the water gradually accumulates towards the downstream embankment. A flow blind spot gradually forms near piles 5, 6, and 7, with no water flow, and the force gradually decreases and approaches zero. However, the force on piles 1, 2, and 4 gradually increases and then reaches the second peak. The force on pile 3 is relatively special. Outside the breach of pile 3, a higher water level is formed due to the accumulation of reflected flow from the downstream dam, resulting in a negative force on pile 3 towards the X-axis. 

**Figure 10:**
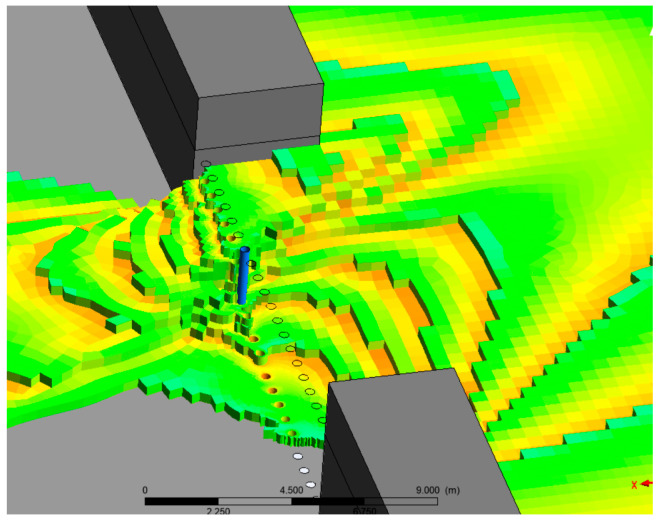
Water flow around the steel pipe pile 3 at T = 13 s.

After the second peak, it gradually enters the steady-state stage. The water flow outside the breach of pile 3 flows towards the downstream dam, and the force on pile 3 gradually tends to be normal. The water flow impacts near pile 1, with high flow velocity and water level. In the steady-state stage, the force is the highest, with a peak force of 3596 N and a final steady-state force of 2400 N. Although the water surface line of pile 2 is higher than that of pile 4, due to the high water level on the outside of the breach, the force in the steady-state stage is smaller than that of pile 4. The steel pipe piles of piles 3, 5, 6, and 7 have relatively small forces in the steady-state stage. From the dynamic time history curve in Figure 9, it can be seen that with the change of time, the root of pile 1 in a single row of piles is under the most stress. The water flow around the pipe pile 3 at T = 13 s is shown in Figure 10.

### 3.4. Analysis of Water Flow Impact Process on Double-Row Steel Pipe Piles

**Figure 11:**
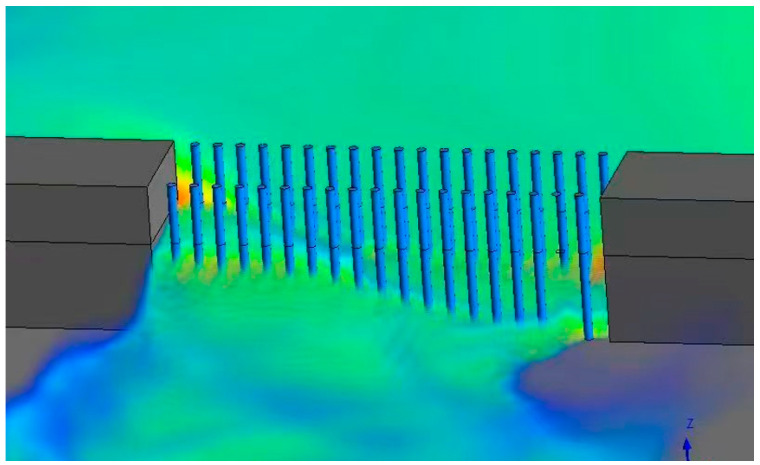
T = 60 s. The volume rendering of water flow through plugging structures.

The arrangement of double-row steel pipe piles is to arrange a row of steel pipe piles on both sides of the breach. The front row piles of double-row steel pipe piles have two functions: first, to reduce the flow velocity of the breach water, reduce the flow rate of the breach, and then reduce the force on the steel pipe piles, ensuring that the force on the rear row steel pipe piles is more uniform. Figure 11 is the schematic diagram of single-row pile breach water flowing through steel pipe piles when T = 60 s.

**Figure 12:**
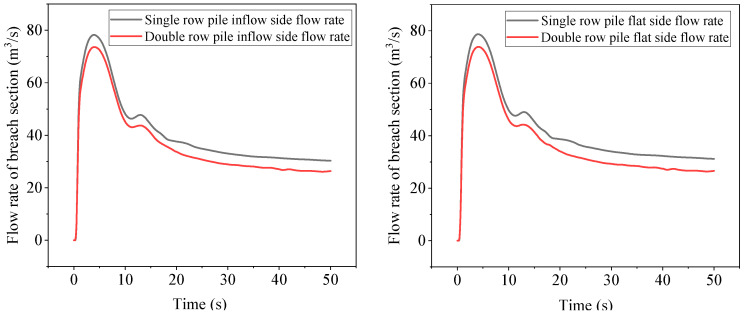
Cross-sectional flow before and after the breach of single- and double-row piles.

Figure 12 shows the comparison of cross-sectional flow rates before and after the collapse of single- and double-row piles. It can be seen from the figure that the flow rate at the collapse first increases rapidly and then decreases rapidly, with a slight increase at T = 11 s, followed by a slow decrease until entering steady state. The flow rate at the collapse of double-row piles is lower than that of single-row piles on both the inflow side and the flat side, with a small decrease in peak force of only 6% and a large decrease of about 13% in steady state. The flow rate at the collapse is an important factor affecting the water surface height and flow velocity of steel pipe piles.

**Figure 13:**
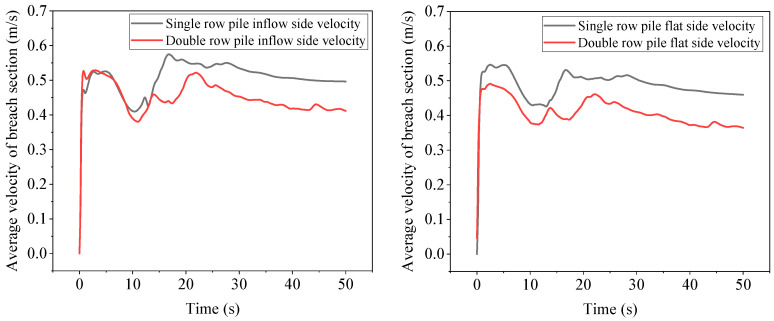
Comparison of average flow velocity before and after breach of single- and double-row piles.

Figure 13 shows the comparison of the average flow velocity before and after the breach of a single-row pile and a double-row pile. Due to the lack of water in most parts of the section, the average flow velocity value is lower. Compared with a single-row pile, the average flow velocity before and after the breach of a double-row pile has significantly decreased. In steady-state, the average flow velocity on the inflow side section decreased by 17.1%, and the average flow velocity on the flat side section decreased by 20.7%. Compared to single-row piles in steady-state. Compared with single-row piles, the decrease in flow rate on the inflow side of double-row piles is smaller, while the decrease in flow rate is larger, indicating that the average water surface line on the inflow side of double-row piles is higher than that of single-row piles. The difference in flow rate between the inflow side and the flat side is relatively small; while comparing the flow rate on the inflow side and flat side, it can be seen that the average flow rate on the flat side of single- and double-row piles has decreased by 7.5% and 11.4%, respectively, compared to the inflow side. It can also be seen that pile arrangement can reduce the flow rate at the breach to a certain extent, but the water surface height will increase after the pile arrangement.

**Figure 14:**
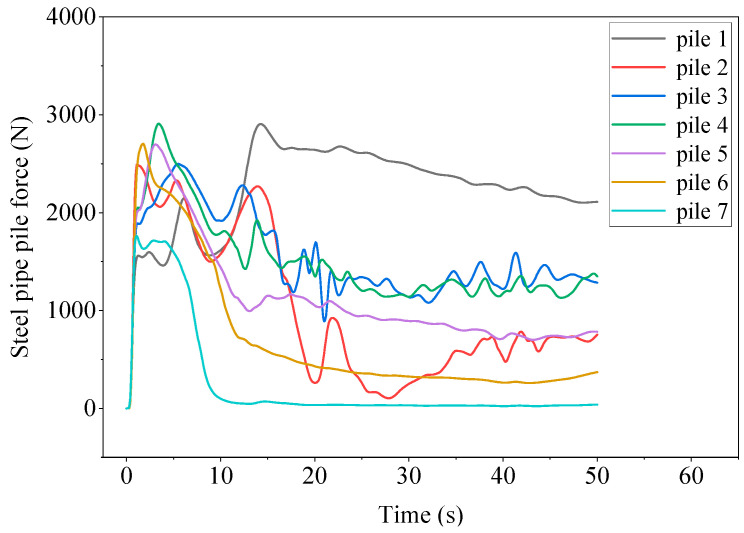
Dynamic time history curve of steel pipe piles 1–7 with double-row piles.

Figure 14 shows the force history curve of the steel pipe piles in the back row from 1 to 7. It can be seen from the graph that compared with single-row piles, the lateral force of double-row piles is more uniform, and the difference in force between different steel pipe piles is reduced. Moreover, all piles are in the positive X-axis direction, and there is no negative force situation. Except for pile 3, the overall force change trend of the other piles is roughly the same as before; in the steady-state stage, the force is not constant but fluctuates within a certain numerical range. In the steady state, the force on pile 1 is the largest, followed by piles 3 and 4, which have similar forces. Then, piles 2 and 5 have similar forces. Pile 6 has a smaller force, while pile 7 has the same force as before. In the steady state, it still approaches 0. When comparing single- and double-row piles in the future, pile 7 will no longer be considered, and the force situation on piles 1–6 will be mainly compared.

In the two-dimensional model time history curve in Figure 13, it can be seen that with the change of time, the pile root of pile 1 in the double-row pile bears the largest stress.

## 4. Result and Discussion

### 4.1. Verification of Numerical Method for Water Flow Impact Force in Plugging Structures

The force situation of the plugging structure in the breach of the embankment was studied through three-dimensional finite element simulation. The Morison equation calculation results based on the numerical model of the breach water flow were verified, and the theoretical calculation results of the water flow impact force were compared to determine whether the values of $C_{D}$ and $C_{M}$ are reasonable.

**Figure 15:**
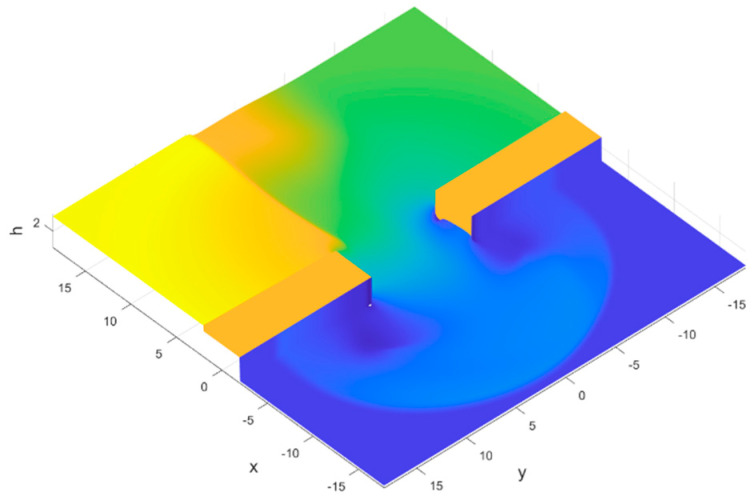
Numerical calculation of water flow situation at embankment breach using MATLAB. The green and yellow color indicates a high water level, while the blue color represents a low water level.

**Figure 16:**
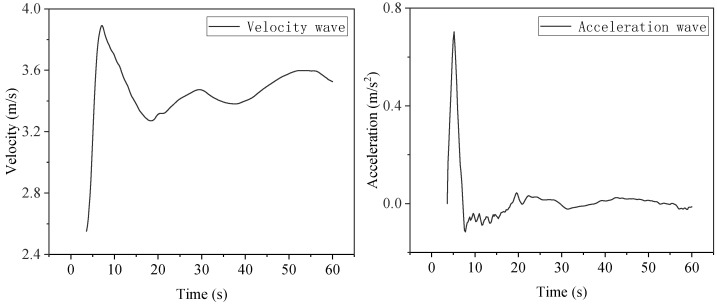
Flow velocity wave and acceleration wave of the breach in front of the plugging structure.

**Figure 17:**
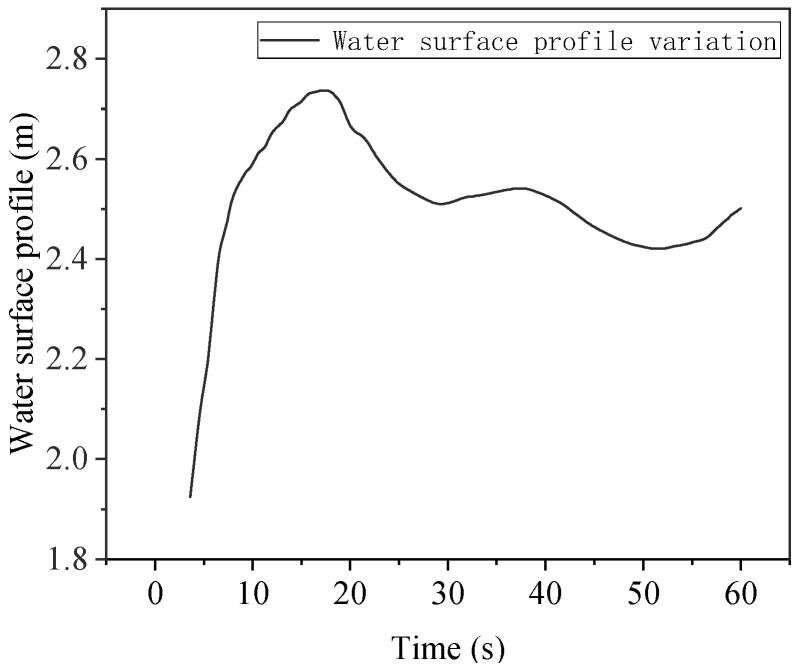
Plugging structural water surface line.

The width of the river is 20 m, the width of the embankment is 4 m, and the width of the breach is 10 m. Under the initial water depth of the river is 3 m, and the initial flow velocity is 3 m/s. The water flow development characteristics of the embankment breach calculated by MATLAB at T = 3 s are shown in Figure 15. The recorded maximum flow velocity in the *X* direction is 4.89 m/s, the maximum flow velocity in the *Y* direction is 3.43 m/s, the maximum average flow velocity in the *X* section at the edge of the breach is 3.89 m/s, and the maximum average horizontal height is 2.71 m. The average velocity wave and acceleration wave in front of the plugging structure in 60 s were calculated using the numerical model, as shown in Figure 16. The average water surface height of the fluid in front of the plugging structure is shown in Figure 17.

According to the derivation in Equation (16), the water flow impact force per unit length of the plugging structure is:

(16) $$F=F_{static}+F_{dynamic}=\int_{0}^{h}\rho gz\times 1\;dz+\int_{0}^{h}\left(C_{D}\times \frac{\rho }{2}{u_{z}}^{2}+C_{M}\times V_{0}\times \rho {\dot{u}}_{z}\right)dz$$

**Figure 18:**
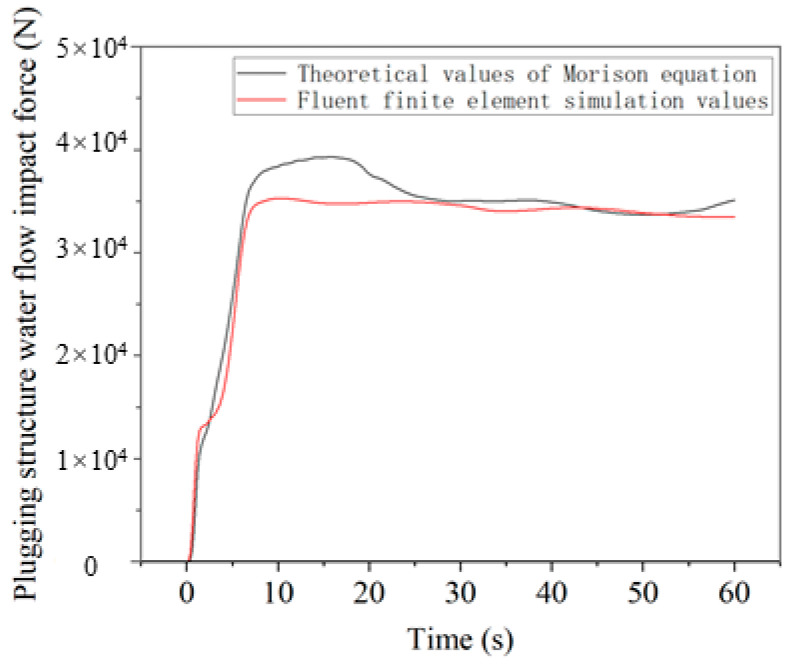
Comparison of theory and simulation.

The theoretical calculation and finite element simulation results comparison of the water flow impact force of the plugging structure are shown in Figure 18. It can be seen from the figure that the overall fit between the theory and the results is good, and the theoretical calculation results are relatively large, with a maximum error of 11.53%. This proves that the theory can calculate the impact force of the plugging structure in the water flow within a certain error range.

### 4.2. Three-Dimensional Simulation Results of the Water Flow Impact Force on the Plugging Structure

#### 4.2.1. At Different Initial Flow Rates

**Figure 19:**
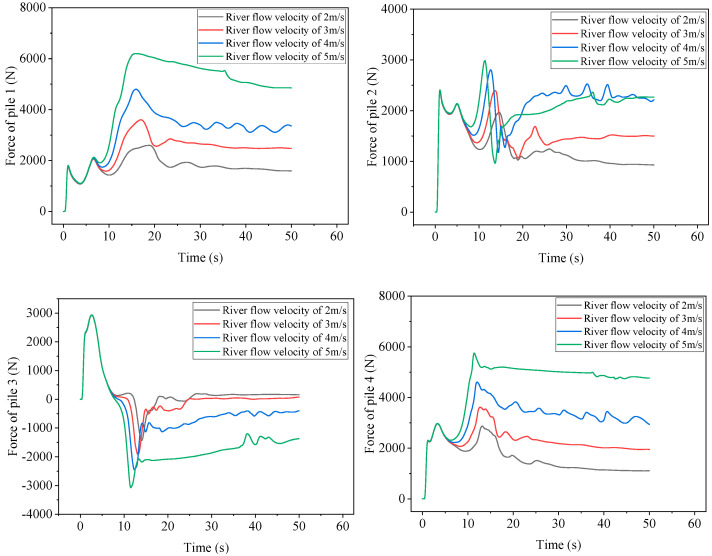
Dynamic time history curves of steel pipe piles 1–4 under different flow velocities.

Through analysis of the force-time history of steel pipe piles 1–7, it is observed that piles 5, 6, and 7 exhibit relatively small peak forces, indicating a need for focused attention on the force of piles 1, 2, 3, and 4. Figure 19 shows the force time history curves of piles 1–4 under different river flow velocities. The higher the river flow velocity, the greater the peak force of pile 1. When the river flow velocity is 5 m/s, the peak force of pile 1 is 6197 N. When the river flow velocity is 2 m/s, the peak force of pile 1 is only 2592 N, which is 2.4 times that of the latter. This is similar to the speed multiplier of the two: the higher the flow velocity of the river, the slower the rate of force reduction of pile 1 after the peak value.

The higher the flow velocity of the river, the earlier and larger the peak time of the second wave of the pile 2, followed by a rapid decrease in force. When the final stage gradually enters a steady state, the performance of the final stage varies under different flow velocities. When the flow velocity of the river is 3 m/s, 4 m/s, and 5 m/s, it first rises and then gradually stabilizes, while the flow velocity of the river is 2 m/s, which gradually decreases and enters a steady state. When the flow velocity of the river is 4 m/s and 5 m/s, the peak force of the entire process of pile 2 appears in the second wave peak and the force during the steady-state stage is almost the same, about 2200 N. When the flow velocity of the river is 2 m/s or 3 m/s, the peak force of the entire process appears at the first wave peak. At a flow velocity of 2 m/s, the steady-state force of pile 2 is about 930 N, and at a flow velocity of 3 m/s, the force is about 1500 N.

The force trend of pile 3 under different flow velocities is basically consistent. When the river flow velocity is high, due to the accumulation of reflected flow downstream of the dam on the outside of the breach, the force in the steady-state stage is still negative in the *X* direction. The higher the flow velocity, the greater the negative force on pile 3. When the river flow velocity is 4 m/s, the force is −400 N, and when the flow velocity is 5 m/s, the force can reach −1370 N. When the river flow velocity is low, the steady-state force is positive, but the absolute value is small.

The force process of pile 4 under different river flow velocities is similar to that of pile 1. Firstly, it rapidly increases to the first peak value; then, it slightly decreases, and then rapidly increases. Different river flow velocities almost reach the second peak force at the same time, and the higher the flow velocity, the higher the second peak force. Subsequently, it gradually decreases and is included in the steady-state stage. There is a significant difference in force during the steady-state stage under different flow velocities. When the river flow velocity is 5 m/s, the force on pile 4 is about four times that of the river flow velocity of 2 m/s, indicating that the river flow velocity has a significant impact on the force on pile 4.

When the water flows around the row piles at a certain speed, the force generated on the row piles can be divided into two directions. One direction is the lateral force formed by the dragging effect of the fluid, which was also discussed earlier; the other part is the lift generated perpendicular to the flow direction, which is the sum of the vertical projections of various small hydrodynamic pressures and small frictional resistance on the surface of the row piles. It is generated due to the unequal flow velocity along the vertical direction of the row piles.

**Figure 20:**
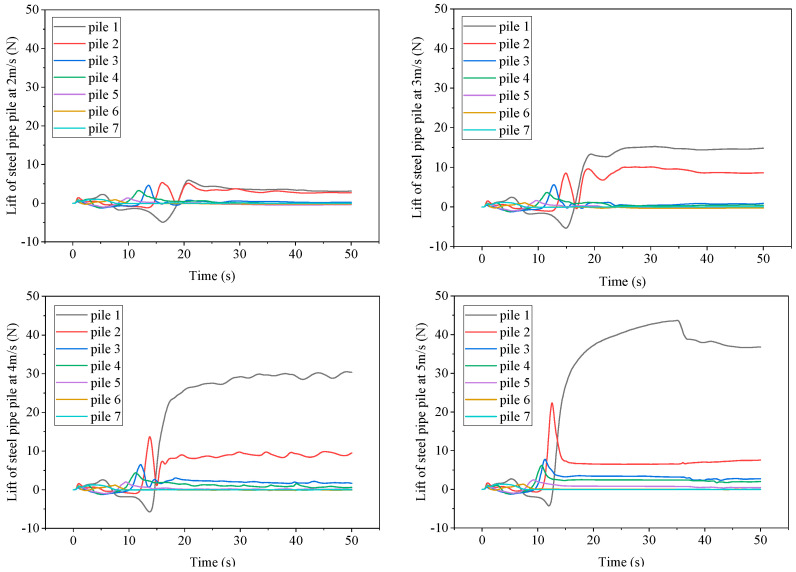
Lift time history curve of steel pipe piles 1–7 under different flow velocities.

Figure 20 shows the lift time history curves of steel pipe piles 1–7 under different flow velocities. It can be seen from the figure that the overall trend of lift changes for piles 1 and 2 is first increasing and then decreasing, followed by a rapid increase and gradually entering a steady state. The rest of the pile positions first increase and then decrease before entering a steady state, and the force in the steady-state stage tends to approach 0. The difference in lift between different piles is significant, mainly because the magnitude of lift is determined by the flow velocity and circulation value. The flow velocity near piles 1 and 2 is high, the flow depth is deep, and the circulation value is large, resulting in a large lift. Piles 3, 4, and 5 only have a large lift in the initial stage. After entering a steady state, the water flows towards the vicinity of pile 1, so the closer the circulation value is to pile 1, the higher the lift. By comparing different flow velocities, the higher the river flow velocity, the greater the lift in the final steady-state stage. When the river flow velocity is 2 m/s, the lift of pile 1 is 3.1 N, and when the river flow velocity is 5 m/s, the lift of pile 1 can reach 36.8 N, which is about 12 times that of the former. As the flow velocity increases, the lift of pile 2 does not change significantly, while the other piles have some changes, but the values are small and tend to approach 0.

#### 4.2.2. At Different Pile Inclination Angles

**Figure 21:**
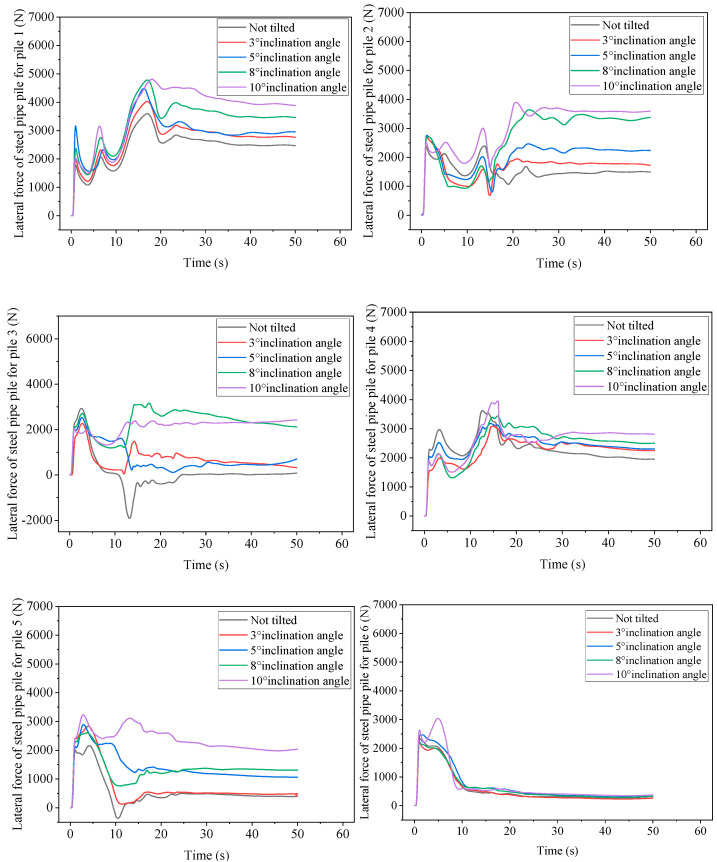
Dynamic time history curves of steel pipe piles 1–6 at different inclination angles.

Due to construction or water flow impact, the plugging process of the embankment breach pile arrangement may cause a certain degree of inclination angle of the pile arrangement. Figure 21 shows the force history curves of steel pipe piles 1–6 at different inclinations, and the force of pile 7 does not show significant changes at different inclinations. Therefore, the force curve of pile 7 was not drawn. As a control study, the peak force of pile 1 appeared at the last wave peak under different inclination angles compared to the non-inclined steel pipe pile. The larger the inclination angle, the greater the peak force. However, the peak forces at an inclination of 10° and 8° were closer, indicating that as the inclination angle increased, the peak force of pile 1 did not always increase. The larger the inclination angle, the more significant the steady-state force is, and the steady-state force increases by 57.03% when inclined at 10° compared to when not inclined. The peak force of the non-inclined pile 2 appears at the second wave peak, while the force on the inclined steel pipe pile will rapidly increase again after the second wave peak decreases. The peak force is at the third wave peak, and then slowly decreases until it enters a steady state. The larger the inclination angle, the greater the peak force, and the greater the force during the steady-state stage. However, the relationship between force and inclination angle is not linear, and inclination has a greater impact on the second pile. When the inclination angle is 10°, the steady-state force of the second pile increases by 140.05% compared to when it is not inclined, and the peak force increases by 63%. In the inclined state, the force on pile 3 is in the positive *X*-axis direction. In the steady-state stage, the larger the inclination angle, the greater the force. The forces on non-inclined, inclined 3°, and inclined 5° piles are closer, but smaller. The forces on inclined 8° and 10° steel pipe piles are closer, much greater than when inclined 5°.

At different inclination angles, the peak force of pile 4 appears in the second wave peak. The force on the non-inclined pile 4 is relatively large, only lower than that at an inclination angle of 10°. In other inclination angles, the greater the inclination angle, the greater the peak force; however, after the second peak, there is a significant decrease in the lateral force of the non-inclined pile. Piles with inclination angles have a smaller decrease in magnitude. In the steady-state stage, it still shows that the larger the inclination angle, the greater the peak force. When the inclination angle is 10°, the steady-state force of pile 4 increases by 44.08% compared to when it is not inclined. The trend and magnitude of the lateral force variation of pile 5 are related to the inclination angle. The peak force of the pile appears at the first peak, with the maximum force at an inclination angle of 10° and the minimum force when not inclined. After the first peak force, the force of the steel pipe pile decreases to the valley bottom, and the larger the inclination angle, the smaller the downward reduction. The force of the steel pipe pile with an inclination angle of 10° only shows a very small decrease, and after reaching the valley bottom, the lateral force gradually increases until it enters a steady state. In the steady-state stage, the larger the inclination angle, the greater the steady-state force on the steel pipe pile. When the inclination angle is 3°, the force on the steel pipe pile is similar to that on the non-inclined steel pipe pile. The steady-state force at other inclination angles has a significant increase, and the steady-state force at an inclination angle of 10° is about five times that of the non-inclined steel pipe pile. The lateral force of pile 6 is almost the same, except for the pile with an inclination angle of 10°, which rises rapidly and then rapidly decreases, then slowly decreases until it enters a steady state. The pile with an inclination angle of 10° shows a second peak after the first peak, slightly higher than the first peak, and then changes consistent with other inclination angle steel pipe piles. The steady-state force of the steel pipe pile of pile 6 is basically the same under different inclination angles, and the value is relatively small.

### 4.3. The Rapid Calculation of Water Flow Impact Force Based on Flow Velocity Sensing Data 

**Figure 22:**
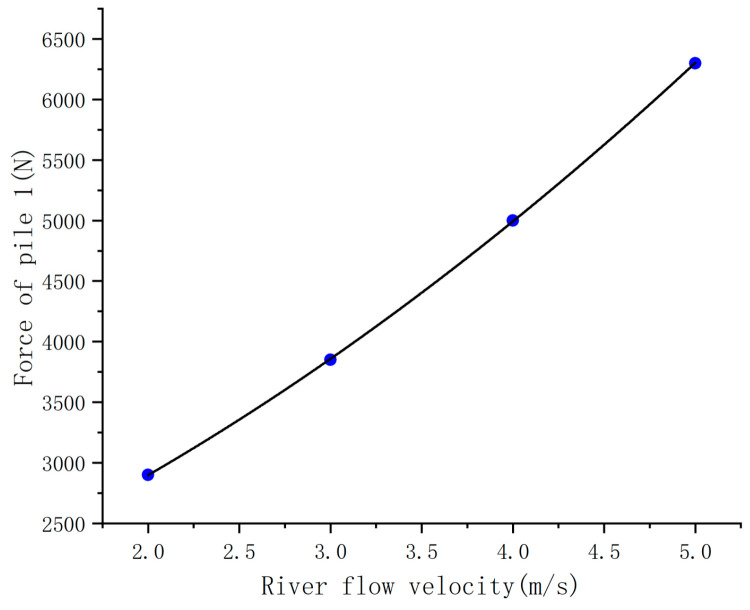
Fitting curve of river flow velocity and pipe pile force.

According to the simulation results, the forces on the pipe piles under different flow velocities can be fitted. As shown in Figure 22, the quadratic polynomial curves are fitted using the forces on pile 1 under flow velocities of 2 m/s, 3 m/s, 4 m/s, and 5 m/s. The formula for this curve is:

(17) $$y=87.5x^{2}+522.5x+1502$$

The sum of squared residuals of the formula is 125, and the R² value is 0.99998. When the flow velocities are 2 m/s, 3 m/s, 4 m/s, and 5 m/s, the errors between the results calculated by the formula and the real results are 0.1%, 0.18%, 0.16%, and 0.03%, respectively, indicating that the fitting effect is very good. 

Through the obtained formula, we can use a velocity sensor to measure the real-time flow velocity of the river and then derive the force on the pipe pile from the flow velocity. This can be used to determine whether the force on the pipe pile exceeds the ultimate bearing capacity.

## 5. Conclusions

This article mainly focuses on the problem of structural force in the plugging of embankment breaches. A theoretical calculation method for the water flow impact force of embankment breach pile arrangement was established by combining the numerical model of breach hydraulics with the Morison equation, and finite element simulation verification was carried out using Fluent. A three-dimensional finite element model considering the coupling effect of water flow and pile arrangement was established, and the effects of flow velocity, water depth, and other factors on the force of the plugging structure were studied. A comparative analysis was conducted on the water flow characteristics and force of single- and double-row piles, and the influence of pile inclination on the force of steel pipe piles was considered. The main conclusions are as follows:

(1) The model was established on a structured grid, the boundary Riemann problem was approximated using the ROE scheme, spatial accuracy was improved using the MUSCL scheme, and the numerical oscillation problem generated by the model was solved by introducing the TVD scheme. Finally, a Godunov scheme hydraulic model was constructed and numerically calculated using MATLAB. The calculation results show that the model can achieve high-precision and oscillation-free numerical calculation of breach flow velocity and water depth.

(2) Based on numerical models, flow velocity waves, acceleration waves, and water surface waves at the breach of embankments were obtained. The Morison equation was derived to calculate the water flow impact force on vertical and inclined piles, and appropriate drag force coefficients *C_D_* and inertia force coefficients *C_M_* were determined. The temporal variation of fluid impact force on plugging structures under different flow velocities and water depths was studied through finite element simulation and theoretical calculations. The research results indicate that the Morison equation method, based on the numerical model, can calculate the impact force of the structure within a certain error range when the value of *C_D_* is set to 1.0 and the value of *C_M_* is set to 2.0, providing a reference for emergency plugging decisions for embankment breaches.

(3) The effect of flow velocity on the maximum force of steel pipe piles is almost linear; double-row piles can reduce the flow rate, flow velocity, and water surface height of the breach to a certain extent, and reduce the force on each steel pipe pile in the rear row. The inclination of pile driving will have a significant impact on the force of steel pipe piles, and the larger the inclination angle, the greater the peak force of steel pipe piles.

## Figures and Tables

**Table 1:** The values of $C_{D}$ and $C_{M}$, recommended by national standards.

National Regulations	Wave Theory	$C_{D}$	$C_{M}$
Hydrological specifications for ports and water-ways (2022 edition) [34]	Linear wave theory	1.2	2.0
American Petroleum Institute Specification (1981 edition) [35]	Stokes’ fifth order wave linear theory	0.6–1.0	1.5–2.0
Rules of the Det Norske Veritas (1974 edition) [36]	Stokes’ fifth order wave theory	0.5–1.2	2.0
China Ship Inspection Bureau Standards (1982 edition) [37]	Stokes’ fifth order wave linear theory	1.0	2.0

## Data Availability

The original contributions presented in the study are included in the article; further inquiries can be directed to the corresponding author.
